# Quantifying Exposure to Wildfire Smoke Among Schoolchildren in California, 2006 to 2021

**DOI:** 10.1001/jamanetworkopen.2023.5863

**Published:** 2023-04-05

**Authors:** Esther E. Velásquez, Tarik Benmarhnia, Joan A. Casey, Rosana Aguilera, Mathew V. Kiang

**Affiliations:** 1 Center for Population Health Sciences, Stanford University School of Medicine, Stanford, California; 2 Scripps Institution of Oceanography, University of California, San Diego; 3 Department of Environmental Health Sciences, Columbia Mailman School of Public Health, New York, New York; 4 Department of Epidemiology and Population Health, Stanford University School of Medicine, Stanford, California

## Abstract

This cross-sectional study quantifies exposure to wildfire particulate matter less than 2.5 μm among schoolchildren in California.

## Introduction

Inhalation of fine particulate matter from wildfire smoke (wildfire particulate matter <2.5 μm in diameter [PM_2.5_]) is detrimental to health, especially for children, who have higher exposure and greater biological susceptibility.^1^ Schools represent an important location for targeted interventions, especially among students from low-income or socially marginalized populations, who are disproportionately affected by asthma and other conditions exacerbated by episodic wildfire PM_2.5_ exposure.^2^ We herein quantify exposure to wildfire PM_2.5_ among schoolchildren in California.

## Methods

In this cross-sectional study, we merged daily zip code–level wildfire PM_2.5_ estimates^3^ with enrollment data from the National Center for Education Statistics for all California public schools for school years ending in 2006 to 2021. We estimated the number of days of wildfire PM_2.5_ exposure greater than 5, 12, and 35 μg/m^3^, weighted by the number of students to estimate the number of student-days of exposure. We performed analyses by self-reported race and ethnicity, school year, and school (eMethods in Supplement 1). Institutional board review and informed consent were not necessary owing to the use of publicly available deidentified data. We followed the STROBE reporting guideline.

## Results

Among the mean (SD) 6 083 428 (134 683) students included, 3 203 351 (137 939) were Hispanic; among non-Hispanic students, 40 698 (7012) were American Indian or Alaska Native, 643 017 (10 669) were Asian, 375 436 (51 615) were Black, and 1 507 678 (129 498) were White. From 2006 to 2021, exposure to wildfire PM_2.5_ greater than 12 μg/m^3^ occurred a mean (SD) of 5 053 262 (8 811 740) student-days per school year. However, the mean masks substantial variation across school years, racial and ethnic groups, and schools. For example, the 2020-2021 school year had the most student-days of wildfire PM_2.5_ greater than 12 μg/m^3^ at over 31 million (Figure, A), and American Indian or Alaska Native children were disproportionately affected with a mean of 9.4 student-days per student compared with the overall mean of 5.5 student-days (Figure, B). That same year, just 15.0% of schools accounted for half of all student-days of wildfire PM_2.5_ greater than 12 μg/m^3^ and just 4.0% accounted for half of all student-days among American Indian or Alaska Native children (Figure, C). During the 2006-2007 school year, 0.5% of schools accounted for 50% of all wildfire PM_2.5_ student-days among American Indian or Alaska Native students. These results remain consistent at other wildfire PM_2.5_ thresholds (Table).

**Figure.**
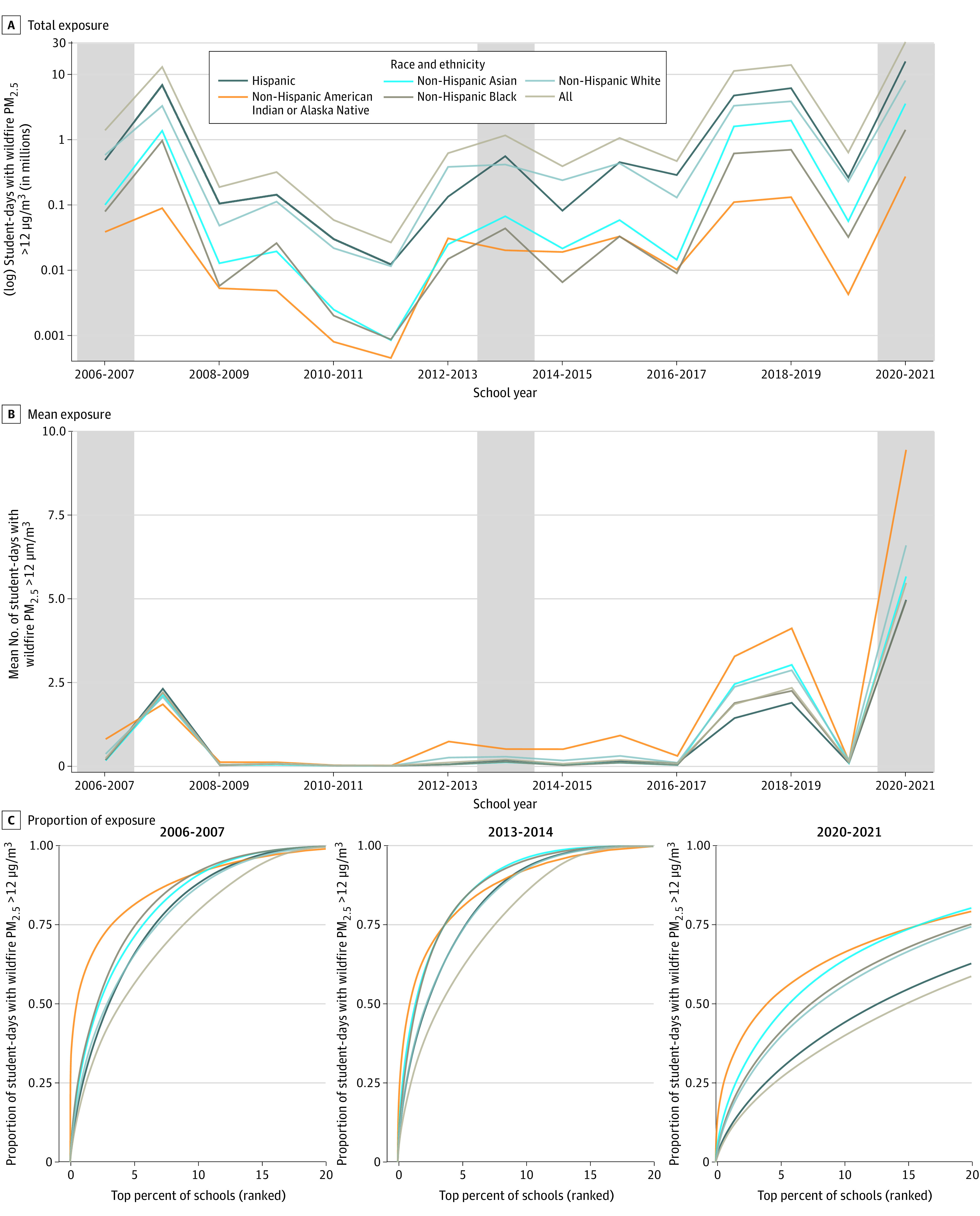
Student-Days of Exposure to Wildfire Particulate Matter Less Than 2.5 μm in Diameter (PM_2.5_) Student-days of wildfire PM_2.5_ exposure are defined as greater than 12 μg/m^3^ and measured by school year and race and ethnicity or over time (A and B). Proportion of student-days of high PM_2.5_ exposure is measured as attributable to the top percentile of schools by race and ethnicity for 3 selected school years (C). The 3 school years highlighted in C are shown with a shaded background in A and B.

**Table.** Student Enrollment and Exposure to Wildfire Particulate Matter <2.5 μm in Diameter in California by Race and Ethnicity, 2006 to 2021^a^

Student race and ethnicity	No. of students enrolled	No. of student-days of exposure to wildfire PM_2.5_ at different thresholds			
		>5 μg/m^3^	>12 μg/m^3^	>35 μg/m^3^
Hispanic	3 203 351 (137 939)	6 401 822 (9 664 935)	2 409 041 (4 393 097)	722 519 (1 530 829)
Non-Hispanic				
American Indian or Alaska Native	40 698 (7012)	116 567 (125 606)	52 203 (74 865)	18 757 (32 921)
Asian	643 017 (10 669)	1 534 245 (2 366 425)	594 795 (1 065 361)	189 744 (411 117)
Black	375 436 (51 615)	707 897 (953 627)	265 549 (448 357)	82 997 (168 664)
White	1 507 678 (129 498)	3 439 945 (4 428 537)	1 415 595 (2 288 710)	454 564 (877 399)
All^b^	6 083 428 (134 683)	12 985 360 (18 732 954)	5 053 262 (8 811 740)	1 578 180 (3 229 438)

Abbreviation: PM_2.5_, fine particulate matter less than 2.5 μm in diameter.

^a^ Data are presented as mean (SD).

^b^ Includes students with unknown, missing, or other race and ethnicity data.

## Discussion

Wildfires and resulting PM_2.5_ are likely to increase in both intensity and frequency due to anthropogenic climate change.^4,5^ There is likely no safe level of exposure to wildfire PM_2.5_, which is particularly hazardous to children and has been linked to reduced lung functioning, asthma, reduced neuropsychological functioning, and increased hospital admissions.^1,6^ Schools offer an ideal site for equitable interventions since children spend much of their time there. While existing interventions focused on individual behavior (eg, masking, reduced outdoor time) may have lower upfront cost, facilities-based interventions (eg, portable air cleaners and central heating, ventilation, and air conditioning) may be more effective and are likely to have higher compliance given their passive mechanisms and provide benefits beyond wildfire PM_2.5_ (eg, ambient PM_2.5_ and filtration of other air pollutants).^1^

This study has important limitations. The use of schools’ outdoor daily wildfire PM_2.5_ exposure as a proxy of children’s total daily exposure ignores children’s exposure at home (which is likely to be inequitable across income and racial and ethnic groups) and outside the school year. Outdoor wildfire PM_2.5_ exposure also ignores previously implemented filtration. Personal measurement to address these weaknesses is an important step for future research. Further, student-level covariates were unavailable.

The findings of this cross-sectional study suggest that prioritizing facilities-based interventions to a small number of schools first may reduce the overall burden of wildfire PM_2.5_ exposure. These policies could include minimum ventilation requirements for new school construction or subsidizing ventilation upgrades for current schools in wildfire-prone areas.

## Notes

Supplement 1. **eMethods.** Analyses by Self-reported Racial and Ethnic Group, School Year, and School
eReferences

Supplement 2.
Data Sharing Statement
