# Supplementary material for: Quantifying Exposure to Wildfire Smoke Among Schoolchildren in California, 2006 to 2021
Source: JAMA Netw Open. 2023 Apr 5;6(4):e235863. doi: 10.1001/jamanetworkopen.2023.5863 (PMC10077104; doi:10.1001/jamanetworkopen.2023.5863)
Supplement: Supplement 1. — eMethods. Analyses by Self-reported Racial and Ethnic Group, School Year, and School eReferences [file jamanetwopen-e235863-s001.pdf]

Supplementary Online Content

Velásquez EE, Benmarhnia T, Casey JA, Aguilera R, Kiang MV. Quantifying exposure to wildfire smoke among schoolchildren in California, 2006 to 2021. *JAMA Netw Open*. 2023;6(4):e235863. doi:10.1001/jamanetworkopen.2023.5863

eMethods. Analyses by Self-reported Racial and Ethnic Group, School Year, and School
eReferences

This supplementary material has been provided by the authors to give readers additional information about their work.

© 2023 Velásquez E et al. *JAMA Network Open*.

eMethods. Analyses by Self-reported Racial and Ethnic Group, School Year, and School

For wildfire PM2.5 exposure, we used a publicly-available data set of average daily wildfire PM2.5 levels at zip code resolution from 2006 through 2021 in the state of California from an ensemble model coupled with spatio-temporal imputation techniques to isolate wildfire smoke specific PM2.5 from ambient PM2.5 (of any source). The data and methods are described in detail elsewhere.[1] For school enrollment, we used publicly available data of public school enrollment by year, grade, race and ethnicity, type of school, and location from the National Center for Education Statistics (NCES).[2] We included students from active public schools in California from kindergarten through high school for school-years ending in 2006-2021. In the NCES data, race and ethnicity are self-reported based on two questions that must be presented to all students (but that students may choose not to answer). First, "Are you Hispanic or Latino" and second, "Select one of more of the following races: American Indian or Alaska Native, Asian, Black or African American, Native Hawaiian or Other Pacific Islander, White." If an individual self-identifies as Hispanic or Latino along with any race category, they are categorized as "Hispanic"; if an individual self-identifies as not Hispanic but only one single race category, they are categorized as that racial group; and lastly, if an individual identifies as not Hispanic and more than one category, they are categorized as "Two or more races".[3]

We assumed a school-year consists of weekdays from August 15 through June 15 of the following year. We removed weekends, winter break (defined as December 20th to January 10th), and the week of Thanksgiving. We excluded virtual schools and adult education programs. For our main analysis, we defined a school day as having "high" wildfire-specific PM2.5 if the value was greater than 12 micrograms per cubic meter in accordance with safety standards set by the US Environmental Protection Agency during the school-year. We calculated school days of high wildfire PM2.5 exposure, weighted by the number of students (i.e., student-days), per school-year overall, by racial and ethnic group, and school. In additional

© 2023 Velásquez E et al. *JAMA Network Open*.

sensitivity analyses, we defined a “high PM2.5 day” as (1) at least 5 micrograms per cubic meter of wildfire-specific PM2.5 and (2) at least 35 micrograms per cubic meter of wildfire-specific PM2.5.

Fully reproducible code and data are available in an online repository at: https://github.com/mkiang/wildfires_school_exposure.

© 2023 Velásquez E et al. *JAMA Network Open*.

eReferences

1. Aguilera R, Luo N, Basu R, et al. A novel ensemble-based statistical approach to estimate daily wildfire-specific PM2.5 in California (2006–2020). Environ Int. 2023;171:107719. doi:10.1016/j.envint.2022.107719

2. U.S. Department of Education. Institute of Education Sciences, National Center for Education Statistics. Available at https://nces.ed.gov/datatools/index.asp?DataToolSectionID=1.

3. National Center for Education Statistics, "Collecting Race and Ethnicity Data from Students and Staff Using the New Categories". https://nces.ed.gov/ipeds/report-your-data/race-ethnicity-collecting-data-for-reporting-purposes

© 2023 Velásquez E et al. *JAMA Network Open*.
