# Supplementary material for: Quantifying Exposure to Wildfire Smoke Among Schoolchildren in California, 2006 to 2021
Source: JAMA Netw Open. 2023 Apr 5;6(4):e235863. doi: 10.1001/jamanetworkopen.2023.5863 (PMC10077104; doi:10.1001/jamanetworkopen.2023.5863)
Supplement: Supplement 2. — Data Sharing Statement [file jamanetwopen-e235863-s002.pdf]

Data Sharing Statement

Velásquez. Quantifying Exposure to Wildfire Smoke Among Schoolchildren in California, 2006 to 2021. *JAMA Netw Open*. Published April 05, 2023.
doi:10.1001/jamanetworkopen.2023.5863

Data
Data available: Yes
Data types: Data (not involving human participants)
How to access data: These data will be publicly available and shared via our Github repository upon publication: https://github.com/mkiang/wildfires_school_exposure. Note that this Github repository is currently private because not all journals allow sharing results before publication. We are happy to share the repository or make it public before publication if the editor wishes.
When available: With publication

Supporting Documents
Document types: Statistical/analytic code
How to access documents: We will make all analytic code publicly available via our Github repository upon publication: https://github.com/mkiang/wildfires_school_exposure. Note that this Github repository is currently private because not all journals allow sharing results before publication. We are happy to share the repository or make it public before publication if the editor wishes.
When available: With publication

Additional Information
Who can access the data: Our data are derived from publicly available sources and will be made available to the public.
Types of analyses: Data will be publicly available for any analyses.
Mechanisms of data availability: These are our data (wildfire PM2.5) or publicly available data (school attendance) and therefore will be made available upon publication.
